# Polycystin‐1 regulates cell proliferation and migration through AKT/mTORC2 pathway in a human craniosynostosis cell model

**DOI:** 10.1111/jcmm.17266

**Published:** 2022-03-13

**Authors:** Maria A. Katsianou, Kostas A. Papavassiliou, Antonios N. Gargalionis, George Agrogiannis, Penelope Korkolopoulou, Dimitrios Panagopoulos, Marios S. Themistocleous, Christina Piperi, Efthimia K. Basdra, Athanasios G. Papavassiliou

**Affiliations:** ^1^ Department of Biological Chemistry Medical School National and Kapodistrian University of Athens Athens Greece; ^2^ First Department of Pathology Medical School National and Kapodistrian University of Athens Athens Greece; ^3^ Department of Neurosurgery Agia Sofia’ Children’s Hospital Athens Greece

**Keywords:** cell migration, cell proliferation, craniosynostosis, mTOR signalling, polycystin‐1

## Abstract

Craniosynostosis is the premature fusion of skull sutures and has a severe pathological impact on childrens’ life. Mechanical forces are capable of triggering biological responses in bone cells and regulate osteoblastogenesis in cranial sutures, leading to premature closure. The mechanosensitive proteins polycystin‐1 (PC1) and polycystin‐2 (PC2) have been documented to play an important role in craniofacial proliferation and development. Herein, we investigated the contribution of PC1 to the pathogenesis of non‐syndromic craniosynostosis and the associated molecular mechanisms. Protein expression of PC1 and PC2 was detected in bone fragments derived from craniosynostosis patients via immunohistochemistry. To explore the modulatory role of PC1 in primary cranial suture cells, we further abrogated the function of PC1 extracellular mechanosensing domain using a specific anti‐PC1 IgPKD1 antibody. Effect of IgPKD1 treatment was evaluated with cell proliferation and migration assays. Activation of PI3K/AKT/mTOR pathway components was further detected via Western blot in primary cranial suture cells following IgPKD1 treatment. PC1 and PC2 are expressed in human tissues of craniosynostosis. PC1 functional inhibition resulted in elevated proliferation and migration of primary cranial suture cells. PC1 inhibition also induced activation of AKT, exhibiting elevated phospho (p)‐AKT (Ser473) levels, but not 4EBP1 or p70S6K activation. Our findings indicate that PC1 may act as a mechanosensing molecule in cranial sutures by modulating osteoblastic cell proliferation and migration through the PC1/AKT/mTORC2 cascade with a potential impact on the development of non‐syndromic craniosynostosis.

## 1 INTRODUCTION

Craniosynostosis refers to unsynchronized ossification of cranial sutures giving rise to both syndromic and non‐syndromic subtypes.^1^ The syndromic subtype is characterized by prematurely fused sutures and several morphological manifestations along with genetic abnormalities. Common symptoms include malformed skull shape, high levels of intracranial pressure, visual or respiratory deficiencies, and neurological dysfunction. It has been linked to various epidemiological factors such as multiple pregnancies, prematurity, birthweight and parents’ age.^2^, ^3^

The non‐syndromic craniosynostosis subtypes account for 70% of the cases and are mainly characterized by fused sutures. Several mutations have been detected in osteoblastogenic genes encoding the fibroblast growth factor receptors (FGFRs), homeobox protein MSX‐2 (MSX2), ephrin‐B (EFNB), twist‐related protein 1 (TWIST1) and runt‐related transcription factor 2 (Runx2). However, the underlying molecular mechanisms remain largely unknown.^4^

Dysfunctional mechanical inputs in the microenvironment contribute to the pathogenesis of craniosynostosis.^5^, ^6^, ^7^ Masticatory forces can induce premature sagittal suture closure in osteopetrotic mice, yet fusion of the internasal suture occurs in mice following soft diet.^5^, ^7^ The presence of extrinsic pathological forces is a contributory factor to premature fusion. Non‐syndromic craniosynostosis has been linked to low foetal station, multiple births and malpresentation. Notably, increased intrauterine forces belong to the common aetiology.^6^

Transient receptor potential channels (TRPs) mediate sensory signals and consist of more than 30 cation channels, which are further subdivided into six subfamilies in mammals: the canonical (TRPC), the vanilloid (TRPV), the melastatin (TRPM), the polycystin (TRPP), the mucolipin (TRPML) and the ankyrin (TRPA) subfamilies.^8^ TRP channels are activated by external or intrinsic stimuli and regulate various physiological and pathological functions. Mutations in *TRP* genes are causative agents in the pathogenesis of TRP channelopathies.^9^ All TRPs seem to have six transmembrane domains, which assemble as homo‐ or hetero‐tetramers within the channel.^10^ Various intracellular and extracellular factors, such as chemical and osmotic stress, trigger the activation of TRPs.^11^

At the extracellular level, TRPs sense signals including chemical, osmotic and mechanical stress.^11^ In several types of cells, they are involved in thermosensation and taste reception.^12^, ^13^ The abundance of intracellular Ca^2+^ stores is sensed by TRPs and thus stimulates signal transduction pathways for the restoration of Ca^2+^ balance. TRPs also contribute to the changes and balance of the concentration of free cytosolic Ca^2+^.^14^ Being located intracellularly or at the plasma membrane, TRPs are also involved in entry and release pathways of Ca^2+^ from cell organelles facilitating its transport.^10^

The mechanosensory molecules and TRP channels, PC1 and PC2, have been implicated in flow mechanosensation, brain injury, skeletal development and osteoblast differentiation.^15^, ^16^, ^17^ Polycystins are expressed in human tissues, including kidneys, blood vessels, pancreas, liver, bone and skull. Being localized at the primary cilium, at the plasma membrane and at the endoplasmic reticulum (ER), they interact with other molecules, connecting the extracellular matrix with the cytoskeleton and thus igniting intracellular signalling pathways.^18^ The intracellular PC1 C‐terminal tail (CT) has been demonstrated to interact and activate several signal transduction pathways including Janus activating kinase (JAK)–signal transducer and activator of transcription (STAT), the mechanistic target of rapamycin (mTOR), Wnt, the activator protein‐1 (AP‐1) and the calcineurin–nuclear factor of activated T‐cell (NFAT) pathways.^18^, ^19^, ^20^

Polycystin‐1‐deficient mice subjected to midpalatal suture expansion and presented craniofacial deformities at the skull base and in craniofacial sutures, a finding which could not be related to signalling mechanisms, though.^16^ Moreover, mutant mice with a conditional deletion of the *polycystic kidney disease 2* (*PKD2*) gene, which encodes for PC2, in neural crest‐derived cells exhibited dysfunctional skull development, such as mechanical trauma, fractured molar roots, distorted incisors, alveolar bone loss and compressed temporomandibular joints, in addition to abnormal skull shapes.^17^

There is also accumulating evidence that mTOR signalling is essential for normal skeletal growth.^21^, ^22^ Discovered in the early 1990s, mTOR is involved in the regulation of essential cell processes.^23^, ^24^, ^25^ A dysfunctional mTOR signalling has been related to various pathogeneses such as cancer and neurodegenerative diseases.^26^, ^27^ More specifically, osteogenesis and craniosynostosis have both been correlated with mTOR signalling.^21^, ^22^ Proliferation and inactivity of stem cells in the adult forebrain are also regulated by mTOR.^28^ The upstream effectors of mTOR, phosphoinositide 3‐kinase (PI3K) and protein kinase B (AKT) are key regulators of the differentiation of various cell types including chondrocytes, osteoblasts, myoblasts and adipocytes.^29^ PI3K acts as a catalyst and results in the production of phosphatidylinositol‐3,4,5‐trisphosphate, activating various signalling components of gene expression and regulators of cell survival.^30^ PI3K is an osteoblast differentiation regulator, interacting with local signalling factors^31^, ^32^, ^33^ and the tissue‐specific Runx2.^34^ PI3K/AKT/mTOR pathway is also involved in the control of the pluripotent stem cells.^35^, ^36^

Since PC1 has previously been shown to induce mTOR signalling and regulate mTOR pathway components activity,^37^, ^38^ we proceeded to investigate the potential implication of PC1/PI3K/AKT/mTOR signalling network in craniosynostosis and its effect on the cell properties of primary cranial suture cells.

## 2 MATERIALS AND METHODS

### 2.1 Tissue samples

The study included 17 suture bone fragments of non‐syndromic craniosynostosis patients (8 with trigonocephaly, 9 patients with dolichocephaly, median age 6 years old, 13 males and 4 females) collected in RNA‐later and formalin at the Department of Neurosurgery of ‘Aghia Sofia’ Children's Hospital, Athens, Greece. The study was approved by the Ethics Committee of the National and Kapodistrian University of Athens (approval number: 1617031071) and ‘Aghia Sofia’ Children's Hospital (approval number: 28749), Athens, Greece. Written informed consent from parents or guardians of children with craniosynostosis was obtained.

### 2.2 Reagents and antibodies

Cell culture media and all tissue culture reagents were obtained from Gibco (ThermoFisher Scientific) and Biosera. For immunohistochemistry, the following reagents were used: Dako Real Envision Detection System, peroxidase/DAB1, rabbit/mouse (Dako). The following primary antibodies were employed for Western blot analysis: polycystin‐2 (sc‐10376, Santa Cruz Biotechnology), p70S6K (sc‐230, Santa Cruz Biotechnology), phospho(p)‐p70S6K (sc‐8416, Santa Cruz Biotechnology), phospho(p)‐mTOR (5536, CST), phospho(p)‐4EBP1 (2855, CST), PTEN (9559,CST), AKT (9272,CST), phospho(p)‐AKT (9271, CST), actin (MAB1501, Millipore), polycystin‐1 CT2741 (kindly provided by the Baltimore Polycystic Kidney Disease Research and Clinical Core Center), mTOR (701483, Thermo Fisher Scientific), anti‐PC1 7E12 (sc‐130554, Santa Cruz Biotechnology) against the extracellular N‐terminal leucine‐rich domain, anti‐PC2 (sc‐28331, Santa Cruz Biotechnology) and anti‐PC2 (PAB2306, Abnova). The following secondary antibodies were used: goat anti‐mouse IgG HRP conjugate (AP124P, Millipore) and goat anti‐rabbit IgG HRP conjugate (AP132P, Millipore). The inhibitory IgPKD1 antibody (blocking the extracellular N‐terminal leucine‐rich domain) was a generous gift from Dr. O. Ibraghimov‐Beskrovnaya and H. Husson (Genzyme), and the PI3K inhibitor was obtained from Cayman, USA.

### 2.3 Primary suture cranial cell cultures

Extracts of human suture tissue from 5 patients (P) with craniosynostosis (3 with trigonocephaly and 2 with dolichocephaly) were isolated by collagenase digestion, and cranial suture cells were cultured according to the methods by Coussens et al.^39^ In brief, the human suture tissue samples were dissected and minced into 1‐mm bone fragments and incubated in 0.25% collagenase for 2 h at 37°C. Samples then were centrifuged, and the supernatant was removed. Samples were then extensively washed with PBS and plated at 5 bone fragments per well, in both 6‐well and 12‐well plates. Cells were cultured in minimal medium in a humidified atmosphere containing 5% CO_2_ kept at 37°C. Minimal medium consisted of aMEM (Gibco, ThermoFisher Scientific), low glucose, supplemented with L‐glutamine, 10% foetal bovine serum (FBS) (Gibco, ThermoFisher Scientific) and 1% antibiotics (penicillin 100 IU/ml, streptomycin 100 μg/ml) (Gibco, ThermoFisher Scientific). Upon confluency, cells were plated in T25 flasks and labelled P1. Medium was changed every 2 days. Cells were passaged to P4 to obtain sufficient amount of cells. All experiments were carried out with cells from the first to the fourth passage after being checked for their osteoblastic characteristics.

### 2.4 Western blotting

Protein extracts were resolved by electrophoresis in SDS‐polyacrylamide gels with varying densities (6% for PC1; 8% for mTOR and p‐mTOR; 10% for p70S6K, p‐p70S6K, AKT, p‐AKT and PTEN; 15% for p‐4EBP1) and transferred to a nitrocellulose membrane (Porablot NCP, Macherey‐Nagel). Membranes were incubated overnight at 4°C with the primary antibodies (dilutions were 1:250 for antibodies against PC1, PC2, mTOR, p70S6K, p‐p70S6K; 1:1000 for p‐mTOR, AKT, p‐AKT, PTEN, p‐4EBP1, actin in PBST containing 1% non‐fat milk). Detection of the immunoreactive bands was performed with the LumiSensor Chemiluminescent HRP Substrate kit (GenScript). Relative protein amounts were evaluated by densitometric analysis using Image J software and normalized to the corresponding actin levels. The experiments were performed in triplicate.

### 2.5 Immunohistochemistry

Paraffin‐embedded tissue specimens were examined by immunostaining employing the two‐step peroxidase‐conjugated polymer technique (Dako Envision) to localize PC1 and PC2, using a solution of Tris/EDTA, pH 9.0 or 6.0 (Target Retrieval Solution 3 in 1, pH 9 10×, Dako, S2367) for all antibodies. The following antibodies were used in these dilutions: anti‐PC1 7E12 (sc‐130554) against the N‐terminal leucine‐rich domain 1:25, anti‐PC2 (PAB2306, Abnova) 1:200. In negative controls, the primary antibody was substituted by nonimmune serum, paraffin‐embedded sections from colorectal cancer were used as positive controls. Sections were incubated with the primary antibodies overnight at 40°C. The expression of polycystin in craniosynostosis samples was calculated as a ratio of positive cells, while the intensity of staining was assessed using the four‐step system: negative =0, weak = +, modest = ++ and strong = +++. The Histo‐score (H‐score) was calculated as the percentage of stained cells multiplied by the staining intensity on a scale from 0 to 300. Immunostaining was evaluated by the pathologists GA and PK.

### 2.6 Cell proliferation assay

Primary cranial suture cells were cultured in 96‐well culture plates. Cell proliferation was assessed by the 2, 3‐Bis‐ (2‐methoxy‐4‐nitro‐5‐sulfophenyl)‐2H‐tetrazolium‐5‐carboxanilide salt) (XTT) method. Full aMEM was replaced with FBS‐free aMEM, and a 24‐h incubation with the inhibitory antibody of the extracellular domain of PC1 (IgPKD1) (anti‐rabbit IgG‐TR (1:50) heeded. XTT reagent (Cayman) was added in a final concentration of 1 mg/ml in aMEM phenol red free (Invitrogen). Then, the cells were incubated for another 2 h at 37°C, and a 450‐nm absorbance was measured.

### 2.7 Cell migration assay

Primary cranial suture cells were cultured in 6‐well culture plates. After cell coating, the cell monolayer was etched with a 200‐μl sterile pipette tip. aMEM was supplemented with (1:50) the inhibitory antibody of the extracellular domain of PC1 (IgPKD1). Each location was photographed in a computer‐connected microscope at 40× and 20× magnifications at the start (0 h) and after 24 h incubation with the inhibitory antibody (1:50). The pictures were analysed by the TScratch software (Wimasis image analysis platform). Results were expressed as per cent of cell‐covered area, wound healing areas taken as wound recovery.

### 2.8 Statistical and image analysis

Statistical analyses were conducted with the SPSS 23.0 and Microsoft Excel software packages. Correlation tests were carried out. All experiments were performed at least three times, and representative results of one experiment are shown. The data are presented as mean  ± SE for the number of experiments indicated and analysed by Student's *t*‐test. All statistical tests were two‐sided. *p*‐values < 0.05 were regarded as statistically significant. The Image J software was used for densitometry quantification analysis.

## 3 RESULTS

### 3.1 Detection and localization of polycystins in human craniosynostosis samples

**FIGURE 1:**
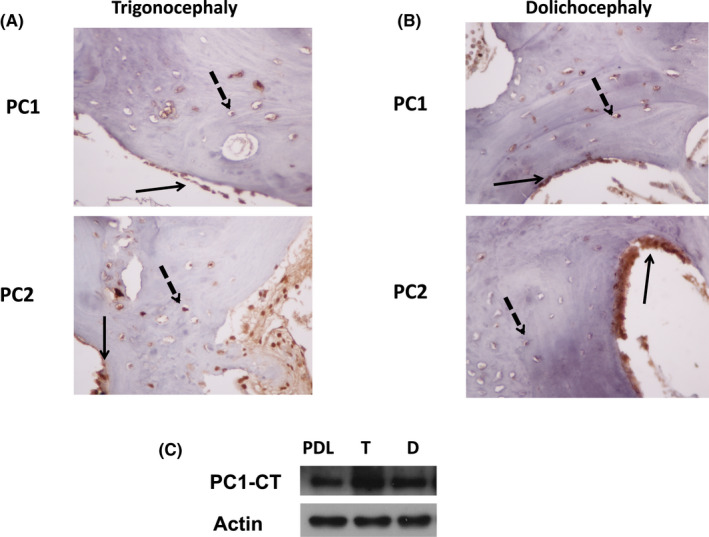
Polycystin‐1/Polycystin‐2 expression levels in human tissues of Trigonocephaly (T) and Dolichocephaly (D) and the respective cranial cells. (A) Immunohistochemical images showing PC1 and PC2 protein expression in human tissues of trigonocephaly, black arrows indicating PC1 and PC2 localization in osteoblasts and dotted black arrows localization in osteocytes respectively. (B) Immunohistochemical images showing PC1 and PC2 protein expression in human tissues of dolichocephaly, black arrows indicating PC1 and PC2 localization in osteoblasts and dotted black arrows localization in osteocytes respectively (400×). Representative images of a total of 17 craniosynostosis patients. (C) Protein expression of PC1 C‐terminal tail in PDL and cranial suture cells T and D

We initially evaluated polycystins’ localization and expression in suture tissue samples by immunohistochemistry. PC1 localization was detected in the cytoplasm of osteoblasts and osteocytes in both craniosynostosis subtypes (Figure 1A,B). PC1 expression in osteoblasts ranged from 0 to 75% (mean value 28%), whereas in osteocytes ranged from 0 to 75% (mean value 33%). The overall H‐score of PC1 ranged from 0 to 225 in osteoblasts and osteocytes with a median up to 25.

Polycystin‐2 localization was also observed in osteoblasts and osteocytes with mostly cytoplasmic expression. PC2 expression ranged from 0 to 100% (mean value 43%), whereas in osteocytes, PC2 ranged from 0 to 75% (mean value 34%). The overall PC2 H‐score in osteoblasts ranged from 0 to 300 with a median of 50. In osteocytes, the median of H‐score was 38, ranging from 0 to 300.

polycystin‐1‐C terminal was identified in primary cranial cells of trigonocephaly and dolichocephaly. Osteoblastic cells of the periodontal ligament (PDL)^20^, ^40^ were used as a positive control (Figure 1C).

### 3.2 PC1 inhibition enhances cell proliferation and migration of primary cranial suture cells

Following the immunohistochemical analysis of tissues samples, we carried on to the isolation and culture of primary suture cells from trigonocephaly and dolichocephaly patients to further explore the potential functional role of PC1 in these cells. The effect of PC1 in cranial cell proliferation was investigated by incubating cells for 24 h with a specific antibody that blocks the extracellular mechanosensing domain of PC1 (IgPKD1) (1:50)^20^ and by monitoring cell proliferation, using XTT assay.

**FIGURE 2:**
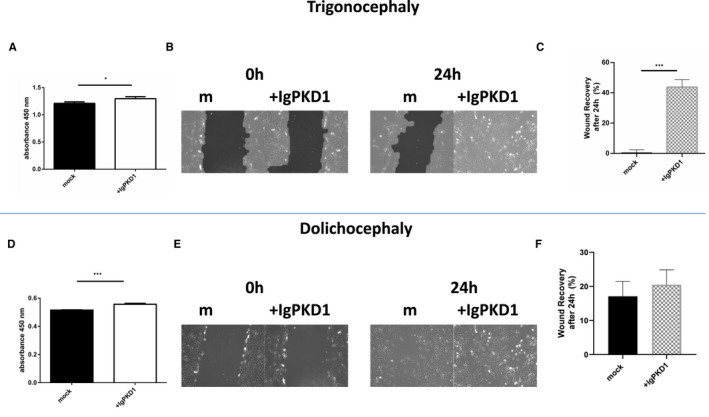
Effect of PC1 functional inhibition on cell proliferation and migration in Trigonocephaly (T) and Dolichocephaly (D) cranial cells. (A) Increased proliferation (*p *< 0.05) of T cranial cells was detected upon treatment of cells with IgPKD1 compared to untreated (mock) cells. (B and C) Increased migration of T cranial cells upon treatment with IgPKD1 for 24 h compared to mock cells (dilution 1:50), *p* < 0.001. The experiments have been performed in triplicate (Student's *t*‐test, *p < 0.05, **p < 0.01, ***p < 0.001). (D) Increased proliferation (*p* < 0.001) of D cranial cells was detected upon treatment of cells with IgPKD1 compared to untreated (mock) cells. (E and F) No difference in migration of D cranial cells upon treatment with IgPKD1 for 24 h compared to mock cells. The experiments have been performed in triplicate (Student's *t*‐test, *p < 0.05, **p < 0.01, ***p < 0.001)

Upon IgPKD1 treatment, trigonocephaly cranial cells exhibited higher proliferation (*p* < 0.05) compared to untreated cells (Figure 2A). Dolichocephaly cells also exhibited increased proliferation (*p* < 0.001) upon PC1 inhibition compared to controls (Figure 2D). The effects of PC1 on cell migration were further studied using the wound healing assay (Figure 2B,E). Trigonocephaly cranial suture cells showed increased migration upon IgPKD1 treatment compared to untreated cells (*p* < 0.001) (Figure 2B,C) whereas dolichocephaly cranial suture cells did not demonstrate such a prominent migratory potential upon IgPKD1 treatment compared to untreated cells (*p* = ns) (Figure 2E,F).

### 3.3 PC1 inhibition induces activation of mTOR pathway components in human primary cranial suture cells

**FIGURE 3:**
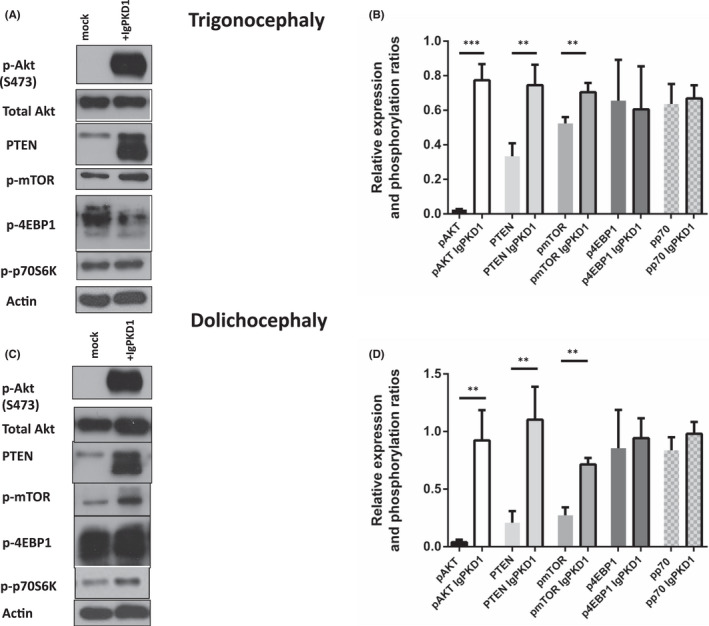
Effect of IgPKD1 treatment on mTOR signalling pathway in Trigonocephaly (T) and Dolichocephaly (D) cranial cells. (A and B) T cranial cells were treated with IgPKD1 (1:50). Western blot analysis of cell lysates of T cranial cells using p‐mTOR, PTEN, p‐AKT, p‐p70S6K and p‐4EBP1 antibodies. Levels of p‐AKT (*p* < 0.001), PTEN (*p* < 0.01) and p‐mTOR (*p* < 0.01) were increased in T cells with IgPKD1 compared to mock cells. pp70S6K and p4EBP1 levels showed *p* = ns. (C and D) D cranial cells were treated with IgPKD1 (1:50). Western blot analysis of cell lysates of D cranial cells using p‐mTOR, PTEN, p‐AKT, p‐p70S6K and p‐4EBP1 antibodies. Levels of p‐AKT (*p* < 0.01), PTEN (*p* < 0.01) and p‐mTOR (*p* < 0.01) were increased in D cells with IgPKD1 compared to mock cells. pp70S6K and p4EBP1 levels showed *p* = ns. The histograms represent densitometry results of the phospho‐immunoblots normalized by total protein levels. Data were analysed by *t*‐test and represent the mean ± SD. The experiments have been performed in triplicate (Student's *t*‐test, *p < 0.05, **p < 0.01, ***p < 0.001). Actin was used as a protein loading control. Representative Western blots are presented

Knowing the association between PC1 and mTOR pathways in other pathophysiologies,^37^, ^38^, ^41^, ^42^ as well as the implication of PI3K/AKT/mTOR pathway in the pathogenesis of craniosynostosis,^21^, ^22^ we aimed to detect any potential interaction of PC1 inhibition with PI3K/AKT/mTOR intracellular signalling cascade in our primary cranial suture cells. In both trigonocephaly and dolichocephaly cranial suture cells, we observed that PC1 inhibition triggered the phosphorylation of AKT (Ser473) and expression of PTEN. The expression of mTOR signalling molecules 4EBP1 and p‐70S6K remained merely unaffected (*p* = ns) (Figure 3A–D).

Interestingly, AKT phosphorylation in Ser473 was absent in trigonocephaly and dolichocephaly untreated cranial suture cells and activated only upon treatment with IgPKD1, indicating a specific effect of PC1 inhibition on these cells. Of note, AKT phosphorylation was present in human chondrocytic ATDC5 or PDL cells, further demonstrating a specific inactivation of AKT in craniosynostotic cells (data not shown).

### 3.4 PC1 inhibition induces specific activation of AKT/mTORC2 signalling in craniosynostosis

The mTOR complex consists of mTOR complex 1 (mTORC1) and mTOR complex 2 (mTORC2). mTORC1 has 4EBP1 and p70S6K among its downstream effectors, while mTORC2 is reciprocally activated with AKT.^43^ Since our findings point towards PC1/AKT/mTORC2 interaction, we examined whether the activation of AKT via mTORC2 in cranial suture cells is PC1‐dependent, by incubating cells with an inhibitor of PI3K/AKT (PI3K103).

**FIGURE 4:**
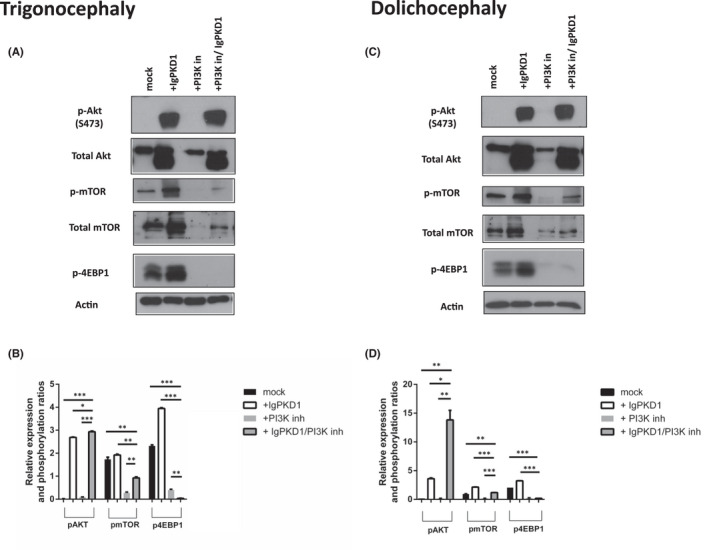
Effect of combined IgPKD1 and PI3K inhibitor treatment on mTOR signalling pathway in Trigonocephaly (T) and Dolichocephaly (D) cranial cells. (A and B) Western blot analysis and quantitative data from cell lysates of T cranial cells using p‐mTOR, p‐AKT and p‐4EBP1 antibodies. (C and D) Western blot analysis and quantitative data from cell lysates of D cranial cells using p‐mTOR, p‐AKT and p‐4EBP1 antibodies. Actin was used as a protein loading control. Representative Western blots of each cell line are presented. All data were analysed by *t*‐test and represent the mean ± SD. The experiments have been performed in triplicate (Student's *t*‐test, *p < 0.05, **p < 0.01, ***p < 0.001)

Immunoblotting analysis showed a notable reduction of p‐mTOR, p‐4EBP1 and p‐AKT expression in PI3K‐inhibited cells in both trigonocephaly and dolichocephaly cranial suture cells. Nevertheless, in cells where IgPKD1 or both inhibitors were present, mTOR and AKT activation recurred. Levels of p‐AKT, p‐mTOR and p4EBP1 were increased in trigonocephaly cells with IgPKD1 compared to mock cells (*p* < 0.001). Levels of p‐AKT, p‐mTOR and p4EBP1 were abrogated with PI3K inhibitor compared to mock (*p* < 0.001) and cells with IgPKD1 inhibitor (*p* < 0.001). The same expression pattern was observed in dolichocephaly cranial suture cells. These data suggest that activation of AKT is PC1‐dependent in treated cells. It can be postulated that PC1 may interact in vitro with the mTOR pathway in craniosynostosis via activation of AKT (Ser 473) (Figure 4A–D).

## 4 DISCUSSION

The elucidation of mechanotransduction pathways and molecular targets that regulate bone remodelling and bone repair in human osteoblastic cells constitutes an ongoing research interest of our laboratory.^20^, ^40^, ^44^, ^45^, ^46^, ^47^ Craniosynostosis is a pathological condition characterized by premature suture closure, ectopic bone formation, and several pathological features including increased ossification, migration and proliferation. Mechanical forces have recently been proposed to contribute to craniosynostosis, indicating mechanosensory proteins and mechanotransduction as important pathogenic mediators.^5^, ^6^, ^48^

Previous studies have demonstrated the implication of mechanosensory polycystins in the cranial development of animal models.^16^, ^17^ In the present study, we investigated for the first time the presence and activity of polycystins in human craniosynostotic tissues and primary cranial suture cells.

We have performed our study on two frequent subtypes of craniosynostosis, namely trigonocephaly and dolichocephaly. Trigonocephaly has been characterized by a pointed and narrow forehead with a triangular shape when viewed from above,^49^ whereas dolichocephaly (positional scaphocephaly) is defined as an elongated anterior–posterior axis of the head as a result of head flattening during side‐to‐side head positioning.^50^

In cranial suture tissue samples of trigonocephaly and dolichocephaly patients, immunohistochemical analysis revealed PC1 and PC2 expression in the cytoplasm of osteoblasts and osteocytes. The PC1 C‐terminal tail (CT) was further detected in both cases at a cellular and protein level, indicating the activation of PC1 and its potential transcriptional role in cranial suture cells along with the ability to interact with intracellular signalling pathways.^18^

Following the original detection of polycystins in tissue samples, we proceeded to investigate the functional role of PC1 in the cellular processes of human primary cranial suture cells. We focused on PC1 because of its main role in mechanosensation, cell‐to‐cell adhesion and cell–matrix interactions.^51^ We have used a well‐characterized antibody (IgPKD1) that binds specifically to the extracellular domain of PC1 and blocks its mechanosensing ability.^20^, ^40^, ^41^, ^42^, ^52^ In this way, PC1 inhibition was shown to promote certain features such as increasing proliferation and migration of cranial cells derived from trigonocephaly and dolichocephaly suture tissues, a finding which was in concert with previous studies, where absent or dysfunctional polycystin exhibited increased proliferation and migration.^40^, ^42^, ^52^

Polycystin‐1 has been implicated in osteogenesis^53^ and in craniofacial development of mouse models.^16^, ^17^ However, its precise role in signalling is not yet fully elucidated in cranial sutures and craniosynostosis. Thus, our next experiments focused on identifying novel interactions in signal transduction pathways. Having identified elevated migration and cell proliferation, we focused on the mTOR signalling pathway as it is connected to PC1 activity and biology of the skeletal tissue.^21^, ^34^, ^37^, ^38^, ^41^, ^42^, ^54^, ^55^, ^56^, ^57^

Mechanistic target of rapamycin is implicated in osteogenesis holding a significant role.^54^ Notably, craniosynostosis experimental studies have detected enhanced mTOR signalling in neural crest cells, which was associated with craniofacial bone lesions.^58^ mTORC1 and mTORC2 are considered as critical regulators of early embryonic development, since disruption of their key components, Raptor or Rictor results in early embryonic death prior to craniofacial organogenesis.^59^, ^60^ Furthermore, mTOR signalling was shown to play a critical role in craniofacial development since mTOR disruption in mesoderm‐derived mesenchymal cells was found to inhibit long bone development and cause calvarial defects.^58^

Of interest, PC1 has been shown to regulate the mTOR signalling pathway in ADPKD studies^37^, ^38^, ^55^, ^57^ with mTORC1 further downregulating the expression of PC1 via a feedback loop.^56^ A previous study from our research team has demonstrated a connection between polycystins and mTOR signalling in colorectal cancer.^42^ In agreement with the above data, we further examined this connection of PC1 and mTOR components in human cranial suture cells. An induction of p‐AKT levels in human cranial suture cells upon PC1 inhibition that was specific to craniosynostotic cells was detected, suggesting its implication in the disease phenotype. To further strengthen this observation, we treated cells with a PI3K inhibitor of the PI3K/AKT/mTOR pathway. In this case, p‐AKT and p‐mTOR were abrogated, an effect that was then reversed when cells were treated with both PI3K and IgPKD1 inhibitors, suggesting that PC1 inhibition can relate to mTOR pathway activation, thus cell migration and proliferation.

Furthermore, we attempted to investigate PTEN expression, which is involved in PI3K/mTOR pathway and is implicated in craniofacial morphogenesis in mice.^61^ We observed increased PTEN levels in cranial suture cells upon PC1 inhibition. PTEN inhibition has previously been demonstrated to modulate PI3K/AKT activity and increase p‐AKT during proliferation of neural crest cells while promoting their differentiation towards osteoblasts.^62^

A recent study has related PTEN to osteogenesis, claiming that high levels of PTEN were responsible for the osteoblastic potential of human dental pulp cells (DP‐MSCs).^63^ In our study, untreated cranial cells present PTEN expression but no AKT phosphorylation, which is in accordance with previous studies indicating that abundant levels of PTEN normally maintain AKT in an inactive form in osteoblastic cells.^64^ However, we identified that upon treatment of cells with PC1 inhibitor, both p‐AKT and PTEN levels were increased, suggesting that PTEN activation may be mediated by another stimulus, other than AKT in suture cells.^65^, ^66^ We suggest that AKT phosphorylation in our study is triggered via mTORC2 as demonstrated by treatment of suture cells with the inhibitor of the PI3K/AKT cascade. Therefore, we pose that diminished PC1‐mediated mechanosensation in cranial suture cells results in the induction of AKT/mTORC2 signalling, sending downstream cues that affect osteogenesis.

## 5 CONCLUSION

In summary, our research highlights the role of PC1 as a regulator of cell proliferation and migration and its interaction with mTOR signalling in human cranial cells. Given that there is a lack of prior research concerning the role of polycystins in craniosynostosis, the present study is one of the first steps towards understanding the function of polycystins in the pathophysiology of craniosynostosis in human conditions.

Future experiments should focus on the mechanism through which PC1 promotes or inhibits cell proliferation and migration, and the molecular details of the interaction between PC1 and mTOR. Moreover, future studies on polycystins and craniosynostosis should explore whether polycystins are associated with any other signalling pathways related to osteogenesis. All the above will contribute to the identification of new prognostic markers in non‐syndromic craniosynostosis as well as to elucidating the key role of PC1 in a new therapeutic scheme against craniosynostosis at a diagnosis level.

Studies on the mechanobiology of craniosynostosis and the respective effect of forces in cranial formation may reveal that craniosynostosis is, in part, a bone condition where mechanical stress and pressure during pregnancy or post‐birth play a crucial role. Such knowledge may advance our understanding of craniosynostosis pathobiology and reveal novel therapeutic targets, presumably using polycystin as a new tool against the development of craniosynostosis.

## CONFLICT OF INTEREST

The authors confirm that there are no conflicts of interest.

## AUTHOR CONTRIBUTIONS

**Maira Katsianou:** Data curation (supporting); Investigation (lead); Methodology (lead); Software (equal); Writing – original draft (equal). **Kostas A. Papavassiliou:** Data curation (equal); Formal analysis (equal); Validation (equal); Writing – original draft (lead); Writing – review & editing (supporting). **Antonios N. Gargalionis:** Formal analysis (supporting); Methodology (supporting); Software (equal); Writing – original draft (supporting). **George Agrogiannis:** Formal analysis (equal); Methodology (equal); Validation (equal). **Penelope Korkolopoulou:** Formal analysis (equal); Methodology (equal); Validation (equal). **Dimitrios Panagopoulos:** Resources (lead). **Marios S. Themistocleous:** Resources (lead). **C. Piperi:** Conceptualization (equal); Data curation (equal); Formal analysis (equal); Project administration (equal); Supervision (equal); Validation (equal). **Efthimia K Basdra:** Conceptualization (lead); Data curation (lead); Formal analysis (lead); Project administration (equal); Supervision (equal); Validation (equal); Visualization (equal). **Athanasios G. Papavassiliou:** Conceptualization (lead); Data curation (lead); Formal analysis (lead); Project administration (lead); Supervision (lead); Validation (lead); Visualization (lead); Writing – review & editing (lead).

## Data Availability

The data that support the findings of this study are available from the corresponding author upon reasonable request.

## References

[1] Beederman M , Farina EM , Reid RR . Molecular basis of cranial suture biology and disease: osteoblastic and osteoclastic perspectives. Genes Dis. 2014;1:120‐125. 25426492 10.1016/j.gendis.2014.07.004 PMC4241362

[2] Lajeunie E , Merrer ML , Bonaïti‐Pellie C , Marchac D , Renier D . Genetic study of nonsyndromic coronal craniosynostosis. Am J Med Genet. 1995;55:500‐504. 7762595 10.1002/ajmg.1320550422

[3] Twigg SR , Wilkie AO . A genetic‐pathophysiological framework for craniosynostosis. Am J Hum Genet. 2015;97:359‐377. 26340332 10.1016/j.ajhg.2015.07.006 PMC4564941

[4] Katsianou MA , Adamopoulos C , Vastardis H , Basdra EK . Signaling mechanisms implicated in cranial sutures pathophysiology: Craniosynostosis. BBA Clin. 2016;6:165‐176. 27957430 10.1016/j.bbacli.2016.04.006 PMC5144105

[5] Oppenheimer AJ , Rhee ST , Goldstein SA , Buchman SR . Force‐induced craniosynostosis in the murine sagittal suture. Plast Reconstr Surg. 2009;124:1840‐1848. 19952640 10.1097/PRS.0b013e3181bf806c PMC3381905

[6] Oppenheimer AJ , Rhee ST , Goldstein SA , Buchman SR . Force‐induced craniosynostosis via paracrine signaling in the murine sagittal suture. J Craniofac Surg. 2012;23:573‐577. 22446418 10.1097/SCS.0b013e318241db3e

[7] Wu BH , Kou XX , Zhang C , et al. Stretch force guides finger‐like pattern of bone formation in suture. PLoS One. 2017;12:e0177159. 28472133 10.1371/journal.pone.0177159 PMC5417680

[8] Corey DP . New TRP channels in hearing and mechanosensation. Neuron. 2003;39:585‐588. 12925273 10.1016/s0896-6273(03)00505-1

[9] Kaneko Y , Szallasi A . Transient Receptor Potential (TRP) channels: a clinical perspective. Br J Pharmacol. 2014;171:2474‐2507. 24102319 10.1111/bph.12414 PMC4008995

[10] Pedersen SF , Owsianik G , Nilius B . TRP channels: an overview. Cell Calcium. 2005;38:233‐252. 16098585 10.1016/j.ceca.2005.06.028

[11] Clapham DE . TRP channels as cellular sensors. Nature. 2003;426:517‐524. 14654832 10.1038/nature02196

[12] Nilius B , Appendino G . Spices: the savory and beneficial science of pungency. Rev Physiol Biochem Pharmacol. 2013;164:1‐76. 23605179 10.1007/112_2013_11

[13] Pedersen SF , Nilius B . Transient receptor potential channels in mechanosensing and cell volume regulation. Methods Enzymol. 2007;428:183‐207. 17875418 10.1016/S0076-6879(07)28010-3

[14] Nilius B , Owsianik G , Voets T , Peters JA . Transient receptor potential cation channels in disease. Physiol Rev. 2007;87:165‐217. 17237345 10.1152/physrev.00021.2006

[15] Boulter C , Mulroy S , Webb S , Fleming S , Brindle K , Sandford R . Cardiovascular, skeletal, and renal defects in mice with a targeted disruption of the Pkd1 gene. Proc Natl Acad Sci USA. 2001;98:12174‐12179. 11593033 10.1073/pnas.211191098 PMC59787

[16] Hou B , Kolpakova‐Hart E , Fukai N , Wu K , Olsen BR . The Polycystic kidney disease 1 (Pkd1) gene is required for the responses of osteochondroprogenitor cells to midpalatal suture expansion in mice. Bone. 2009;44:1121‐1133. 19264154 10.1016/j.bone.2009.02.018 PMC2680722

[17] Kolpakova‐Hart E , McBratney‐Owen B , Hou B , et al. Growth of cranial synchondroses and sutures requires polycystin‐1. Dev Biol. 2008;321:407‐419. 18652813 10.1016/j.ydbio.2008.07.005 PMC2576296

[18] Retailleau K , Duprat F . Polycystins and partners: proposed role in mechanosensitivity. J Physiol. 2014;592:2453‐2471. 24687583 10.1113/jphysiol.2014.271346 PMC4080931

[19] Dalagiorgou G , Basdra EK , Papavassiliou AG . Polycystin‐1: function as a mechanosensor. Int J Biochem Cell Biol. 2010;42:1610‐1613. 20601082 10.1016/j.biocel.2010.06.017

[20] Dalagiorgou G , Piperi C , Georgopoulou U , Adamopoulos C , Basdra EK , Papavassiliou AG . Mechanical stimulation of polycystin‐1 induces human osteoblastic gene expression via potentiation of the calcineurin/NFAT signaling axis. Cell Mol Life Sci. 2013;70:167‐180. 23014991 10.1007/s00018-012-1164-5 PMC5012114

[21] Chen J , Long F . mTOR signaling in skeletal development and disease. Bone Res. 2018;6:1. 29423330 10.1038/s41413-017-0004-5 PMC5802487

[22] Linder M , Hecking M , Glitzner E , et al. EGFR controls bone development by negatively regulating mTOR‐signaling during osteoblast differentiation. Cell Death Differ. 2018;25:1094‐1106. 29445126 10.1038/s41418-017-0054-7 PMC5988706

[23] Brown EJ , Albers MW , Bum Shin T , et al. A mammalian protein targeted by G1‐arresting rapamycin‐receptor complex. Nature. 1994;369:756‐758. 8008069 10.1038/369756a0

[24] Laplante M , Sabatini DM . mTOR signaling in growth control and disease. Cell. 2012;149:274‐293. 22500797 10.1016/j.cell.2012.03.017 PMC3331679

[25] Sabers CJ , Martin MM , Brunn GJ , et al. Isolation of a protein target of the FKBP12‐rapamycin complex in mammalian cells. J Biol Chem. 1995;270:815‐822. 7822316 10.1074/jbc.270.2.815

[26] Maiese K . Targeting molecules to medicine with mTOR, autophagy and neurodegenerative disorders. Br J Clin Pharmacol. 2016;82:1245‐1266. 26469771 10.1111/bcp.12804 PMC5061806

[27] Sabatini DM . mTOR and cancer: insights into a complex relationship. Nat Rev Cancer. 2006;6:729‐734. 16915295 10.1038/nrc1974

[28] Paliouras GN , Hamilton LK , Aumont A , Joppe SE , Barnable‐Heider F , Fernandes KJ . Mammalian target of rapamycin signaling is a key regulator of the transit‐amplifying progenitor pool in the adult and aging forebrain. J Neurosci. 2012;32:15012‐15026. 23100423 10.1523/JNEUROSCI.2248-12.2012 PMC6704835

[29] Hidaka K , Kanematsu T , Takeuchi H , Nakata M , Kikkawa U , Hirata M . Involvement of the phosphoinositide 3‐kinase/protein kinase B signaling pathway in insulin/IGF‐I‐induced chondrogenesis of the mouse embryonal carcinoma‐derived cell line ATDC5. Int J Biochem Cell Biol. 2001;33:1094‐1103. 11551825 10.1016/s1357-2725(01)00067-x

[30] Downward J . PI 3‐kinase, Akt and cell survival. Semin Cell Dev Biol. 2004;15:177‐182. 15209377 10.1016/j.semcdb.2004.01.002

[31] Debiais F , Lefevre G , Lemonnier J , et al. Fibroblast growth factor‐2 induces osteoblast survival through a phosphatidylinositol 3‐kinase‐dependent, ‐beta‐catenin‐independent signaling pathway. Exp Cell Res. 2004;297:235‐246. 15194439 10.1016/j.yexcr.2004.03.032

[32] Golden LH , Insogna KL . The expanding role of PI3‐kinase in bone. Bone. 2004;34:3‐12. 14751558 10.1016/j.bone.2003.09.005

[33] Radcliff K , Tang TB , Lim J , et al. Insulin‐like growth factor‐I regulates proliferation and osteoblastic differentiation of calcifying vascular cells via extracellular signal‐regulated protein kinase and phosphatidylinositol 3‐kinase pathways. Circ Res. 2005;96:398‐400. 15692088 10.1161/01.RES.0000157671.47477.71

[34] Fujita T , Azuma Y , Fukuyama R , et al. Runx2 induces osteoblast and chondrocyte differentiation and enhances their migration by coupling with PI3K‐Akt signaling. J Cell Biol. 2004;166:85‐95. 15226309 10.1083/jcb.200401138 PMC2172136

[35] Singh RP , Dhariwal D , Bhujel N , et al. Role of parental risk factors in the aetiology of isolated non‐syndromic metopic craniosynostosis. Br J Oral Maxillofac Surg. 2010;48:438‐442. 20510490 10.1016/j.bjoms.2009.06.233

[36] Yu JS , Cui W . Proliferation, survival and metabolism: the role of PI3K/AKT/mTOR signalling in pluripotency and cell fate determination. Development. 2016;143:3050‐3060. 27578176 10.1242/dev.137075

[37] Dere R , Wilson PD , Sandford RN , Walker CL . Carboxy terminal tail of polycystin‐1 regulates localization of TSC2 to repress mTOR. PLoS One. 2010;5:e9239. 20169078 10.1371/journal.pone.0009239 PMC2821926

[38] Distefano G , Boca M , Rowe I , et al. Polycystin‐1 regulates extracellular signal‐regulated kinase‐dependent phosphorylation of tuberin to control cell size through mTOR and its downstream effectors S6K and 4EBP1. Mol Cell Biol. 2009;29:2359‐2371. 19255143 10.1128/MCB.01259-08 PMC2668371

[39] Coussens AK , Hughes IP , Morris CP , Powell BC , Anderson PJ . In vitro differentiation of human calvarial suture derived cells with and without dexamethasone does not induce in vivo‐like expression. J Cell Physiol. 2009;218:183‐191. 18803234 10.1002/jcp.21586

[40] Katsianou M , Papavassiliou KA , Zoi I , et al. Polycystin‐1 modulates RUNX2 activation and osteocalcin gene expression via ERK signalling in a human craniosynostosis cell model. J Cell Mol Med. 2021;25:3216‐3225. 33656806 10.1111/jcmm.16391 PMC8034462

[41] Dalagiorgou G , Piperi C , Adamopoulos C , et al. Mechanosensor polycystin‐1 potentiates differentiation of human osteoblastic cells by upregulating Runx2 expression via induction of JAK2/STAT3 signaling axis. Cell Mol Life Sci. 2017;74:921‐936. 27699453 10.1007/s00018-016-2394-8 PMC11107574

[42] Gargalionis AN , Korkolopoulou P , Farmaki E , et al. Polycystin‐1 and polycystin‐2 are involved in the acquisition of aggressive phenotypes in colorectal cancer. Int J Cancer. 2015;136:1515‐1527. 25123959 10.1002/ijc.29140

[43] Gargalionis AN , Malakou LS , Adamopoulos C , et al. Polycystin‐1 downregulation induces ERK‐dependent mTOR pathway activation in a cellular model of psoriasis. Biochim Biophys Acta Mol Basis Dis. 2018;1864:3468‐3476. 30077613 10.1016/j.bbadis.2018.07.036

[44] Fu W , Hall MN . Regulation of mTORC2 signaling. Genes. 2020;11:9. 10.3390/genes11091045 PMC7564249 32899613

[45] Karamesinis K , Spyropoulou A , Dalagiorgou G , et al. Continuous hydrostatic pressure induces differentiation phenomena in chondrocytes mediated by changes in polycystins, SOX9, and RUNX2. J Orofac Orthop. 2017;78:21‐31. 27909759 10.1007/s00056-016-0061-1

[46] Papachroni KK , Karatzas DN , Papavassiliou KA , Basdra EK , Papavassiliou AG . Mechanotransduction in osteoblast regulation and bone disease. Trends Mol Med. 2009;15:208‐216. 19362057 10.1016/j.molmed.2009.03.001

[47] Ziros PG , Gil A‐P , Georgakopoulos T , et al. The bone‐specific transcriptional regulator Cbfa1 is a target of mechanical signals in osteoblastic cells. J Biol Chem. 2002;277:23934‐23941. 11960980 10.1074/jbc.M109881200

[48] Davis C , Lauritzen CGK . The biomechanical characteristics of cranial sutures are altered by spring cranioplasty forces. Plast Reconstr Surg. 2010;125:1111‐1118. 20335863 10.1097/PRS.0b013e3181d0abcf

[49] Governale LS . Craniosynostosis. Pediatr Neurol. 2015;53:394‐401. 26371995 10.1016/j.pediatrneurol.2015.07.006

[50] Allanson JE , Cunniff C , Hoyme HE , McGaughran J , Muenke M , Neri G . Elements of morphology: standard terminology for the head and face. Am J Med Genet A. 2009;149A:6‐28. 19125436 10.1002/ajmg.a.32612 PMC2778021

[51] Kim E , Arnould T , Sellin LK , et al. The polycystic kidney disease 1 gene product modulates Wnt signaling. J Biol Chem. 1999;274:4947‐4953. 9988738 10.1074/jbc.274.8.4947

[52] Ibraghimov‐Beskrovnaya O , Bukanov NO , Donohue LC , Dackowski WR , Klinger KW , Landes GM . Strong homophilic interactions of the Ig‐like domains of polycystin‐1, the protein product of an autosomal dominant polycystic kidney disease gene, PKD1. Hum Mol Genet. 2000;9:1641‐1649. 10861291 10.1093/hmg/9.11.1641

[53] Katsianou MA , Skondra FG , Gargalionis AN , Piperi C , Basdra EK . The role of transient receptor potential polycystin channels in bone diseases. Ann Transl Med. 2018;6:246. 30069448 10.21037/atm.2018.04.10 PMC6046287

[54] Chen J , Long F . mTORC1 signaling controls mammalian skeletal growth through stimulation of protein synthesis. Development. 2014;141:2848‐2854. 24948603 10.1242/dev.108811 PMC4197614

[55] Mekahli D , Decuypere JP , Sammels E , et al. Polycystin‐1 but not polycystin‐2 deficiency causes upregulation of the mTOR pathway and can be synergistically targeted with rapamycin and metformin. Pflugers Arch. 2014;466:1591‐1604. 24193408 10.1007/s00424-013-1394-x

[56] Pema M , Drusian L , Chiaravalli M , et al. mTORC1‐mediated inhibition of polycystin‐1 expression drives renal cyst formation in tuberous sclerosis complex. Nat Commun. 2016;7:10786. 26931735 10.1038/ncomms10786 PMC4778067

[57] Shillingford JM , Murcia NS , Larson CH , et al. The mTOR pathway is regulated by polycystin‐1, and its inhibition reverses renal cystogenesis in polycystic kidney disease. Proc Natl Acad Sci USA. 2006;103:5466‐5471. 16567633 10.1073/pnas.0509694103 PMC1459378

[58] Kramer K , Yang J , Swanson WB , et al. Rapamycin rescues BMP mediated midline craniosynostosis phenotype through reduction of mTOR signaling in a mouse model. Genesis. 2018;56:e23220. 30134066 10.1002/dvg.23220 PMC6108447

[59] Guertin DA , Stevens DM , Thoreen CC , et al. Ablation in mice of the mTORC components raptor, rictor, or mLST8 reveals that mTORC2 is required for signaling to Akt‐FOXO and PKCalpha, but not S6K1. Dev Cell. 2006;11:859‐871. 17141160 10.1016/j.devcel.2006.10.007

[60] Shiota C , Woo JT , Lindner J , Shelton KD , Magnuson MA . Multiallelic disruption of the rictor gene in mice reveals that mTOR complex 2 is essential for fetal growth and viability. Dev Cell. 2006;11:583‐589. 16962829 10.1016/j.devcel.2006.08.013

[61] Yang T , Moore M , He F . Pten regulates neural crest proliferation and differentiation during mouse craniofacial development. Dev Dyn. 2018;247:304‐314. 29115005 10.1002/dvdy.24605 PMC5771892

[62] Guntur AR , Reinhold MI , Cuellar J , Naski MC . Conditional ablation of Pten in osteoprogenitors stimulates FGF signaling. Development. 2011;138:1433‐1444. 21385768 10.1242/dev.058016 PMC3050668

[63] Shen WC , Lai YC , Li LH , et al. Methylation and PTEN activation in dental pulp mesenchymal stem cells promotes osteogenesis and reduces oncogenesis. Nat Commun. 2019;10:2226. 31110221 10.1038/s41467-019-10197-x PMC6527698

[64] Wang H , Sun W , Ma J , Pan Y , Wang L , Zhang W . Polycystin‐1 mediates mechanical strain‐induced osteoblastic mechanoresponses via potentiation of intracellular calcium and Akt/beta‐catenin pathway. PLoS One. 2014;9:e91730. 24618832 10.1371/journal.pone.0091730 PMC3950298

[65] Moutsatsou P , Papavassiliou AG . The glucocorticoid receptor signalling in breast cancer. J Cell Mol Med. 2008;12:145‐163. 18053085 10.1111/j.1582-4934.2007.00177.x PMC3823477

[66] Stechschulte LA , Wuescher L , Marino JS , Hill JW , Eng C , Hinds TD . Glucocorticoid receptor β stimulates Akt1 growth pathway by attenuation of PTEN. J Biol Chem. 2014;289:17885‐17894. 24817119 10.1074/jbc.M113.544072 PMC4067219

